# Assessing the quality of radiation oncology education in four German medical schools: a student perspective and comparison with DEGRO recommendations

**DOI:** 10.1007/s00066-025-02442-8

**Published:** 2025-07-21

**Authors:** Philipp Linde, Frauke Lang, Christiane Matuschek, Marsha Schlenter, Davide Scafa, Judith Neuwahl, Matthias Mäurer, Michael Oertel, Hendrik Dapper, Emmanouil Fokas, Marie Klein, Christian Baues

**Affiliations:** 1 Department of Radiation Oncology, Cyberknife and Radiation Therapy, Faculty of Medicine and University Hospital of Cologne, University of Cologne, Kerpener Str. 62, 50937 Cologne, Germany; 2 Medical School and University Medical Center OWL, Klinikum Mitte, Department of Radiation Oncology, Bielefeld University, Bielefeld, Germany; 3 Department of Radiation Oncology, RWTH Aachen University, Aachen, Germany; 4 Department of Radiation Oncology, University Hospital Bonn, Bonn, Germany; 5 Clinic and Policlinic for Radiation Therapy and Radiooncology, Medical Faculty and University Hospital Düsseldorf, Heinrich-Heine-University Düsseldorf, Düsseldorf, Germany; 6 Department of Radiation Oncology, Jena University Hospital, Jena, Germany; 7 Department of Radiation Oncology, University Hospital Muenster, Muenster, Germany; 8 Department of Radiation Oncology, Marienhospital Herne, Ruhr University Bochum, Bochum, Germany

**Keywords:** Radiotherapy, Medical school, Career, Students’ opinion, Medical studies

## Abstract

**Purpose:**

The increasing importance of radiation oncology (RO) education worldwide drives a need for modernization of university curricula, particularly in anticipation of the new Licensing Regulation for Physicians (*Ärztliche Approbationsordnung*, ÄApprO) in Germany. This study evaluates RO education at four German medical schools and compares student perspectives with the recommendations of the academic radiation oncology consortium of the German Society of Radiation Oncology (*Deutsche Gesellschaft für Radioonkologie*, DEGRO) in order to highlight gaps and opportunities for curriculum improvement.

**Methods:**

A cross-sectional survey was conducted among medical students from the universities in Aachen, Bonn, Cologne, and Düsseldorf (ABCD) from January to June 2022. The standardized digital questionnaire included 31 questions (Likert scale and open-ended items) assessing teaching quality, learning formats, and practical relevance. Descriptive and thematic analyses were performed.

**Results:**

A total of 152 fully completed surveys were analyzed. Most students (76%) reported the use of clinical case examples, but only 13% had direct patient contact. Small-group teaching and hybrid learning formats were preferred. ABCD students emphasized the need for clearer learning objectives and improved differentiation of RO from related disciplines. While 50% supported increased semester hours, opinions were divided. Findings were largely consistent with DEGRO’s recommendations, though discrepancies were observed in the organization of RO within medical curricula.

**Conclusion:**

This study identified key areas for improving RO education, including clearer learning objectives, more interactive and clinically relevant teaching, and increased opportunities for hands-on experience. Integrating these student-informed recommendations into future curriculum reforms may enhance training quality, promote engagement, and support interest in RO as a career.

## Introduction

The rapid advancements in science and technology, along with the growing global burden of cancer, underscore the urgent need to keep radiation oncology (RO) education up to date and to continuously improve it. In 2020, approximately 10 million cancer-related deaths occurred worldwide, with projections indicating a 50% increase by 2040 [1]. While surgery was historically the primary treatment, RO, alongside surgery and chemotherapy, now forms a core pillar of modern oncology care [2]. Radiation oncology is administered to 50–67% of cancer patients, significantly improving outcomes across various malignancies [3, 4]. Consequently, the demand for trained radiation oncologists continues to rise, emphasizing the need for structured and standardized education [5, 6].

Radiation oncology is an interdisciplinary field, integrating theoretical knowledge (radiation biology, physics) with clinical skills (patient interaction, treatment planning) [7]. However, RO curricula in Germany lack uniformity. While some faculties teach it as an independent subject, the majority (62%) integrate it into interdisciplinary modules alongside radiology, nuclear medicine, and pathology [8]. This variation highlights the need for standardization to ensure comprehensive training across institutions and to inspire medical students to consider a career in RO [9].

Medical education has increasingly shifted from traditional knowledge-based models to competency-based medical education (CBME), emphasizing clinical problem-solving and practical skills [10–12]. In this context, and in anticipation of the new Licensing Regulation for Physicians (*Ärztliche Approbationsordnung*, ÄApprO) in Germany, problem-based learning (PbL) has emerged as a preferred method, allowing students to develop clinical reasoning through case-based discussions [13, 14]. A study at the Department of Radiation Oncology, CyberKnife, and Radiation Therapy at the Medical Faculty and University of Cologne (UoC) confirmed that students favor PbL over conventional lectures in RO education [15].

The COVID-19 pandemic accelerated the adoption of digital learning, transforming medical education globally [16]. While online learning offers flexibility, concerns regarding reduced interactivity and limited hands-on training persist [17, 18]. Studies indicate that e‑learning is as effective as traditional teaching, with RO students showing high receptivity to digital lectures [19, 20]. A hybrid model combining in-person and digital formats appears to be a sustainable approach that students may prefer [17, 21].

To address evolving educational needs, Germany’s Masterplan for Medical Education (*Masterplan Medizinstudium*) aims to standardize medical curricula by implementing competency-driven, research-based learning models, optimistically by 2027 at the earliest [22, 23]. The reform allocates 75% core curriculum content with 25% faculty-specific flexibility [24]. In addition, the updated National Competency-Based Learning Objectives for Medicine (NKLM 2.0) emphasize oncology and interdisciplinary competencies in undergraduate education [25]. In response, the German Society of Radiation Oncology (*Deutsche Gesellschaft für Radioonkologie*, DEGRO) proposed 25 compulsory teaching units, focusing on seminars and practical training with direct patient contact [26].

However, these recommendations were developed without direct student input, despite their role as the primary recipients of RO education. Following Kern et al.’s curriculum development model, assessing student perspectives is crucial for optimizing instructional design [27]. This study addresses that need by comparing DEGRO’s recommendations with student perspectives from four German universities (Aachen, Bonn, Cologne, Düsseldorf; ABCD). By gathering student feedback, this study aims to inform curriculum development, focusing on teaching quality, instructional methods, the impact of digital education, and student preferences for additional RO instruction. Additionally, it evaluates different teaching models to identify improvements ensuring RO education aligns with both professional standards and student needs.

## Methods

### Study design and procedures

This prospective cross-sectional study was conducted using a non-probabilistic sample with the primary objective of evaluating the quality of RO education across four German medical universities and their associated hospitals: Aachen (A), Bonn (B), Cologne (C), and Düsseldorf (D). A standardized digital questionnaire was administered between January 1, 2022, and June 30, 2022, via the survey software LimeSurvey (version 5.4.1, LimeSurvey GmbH, Hamburg, Germany). The survey adhered to the General Data Protection Regulation (GDPR) guidelines set by the UoC. To ensure participant anonymity, no personal identification (ID; e.g., names, student IDs, email addresses) was collected. All responses were pseudonymized, and automatically generated IDs were assigned to prevent re-identification. Participation was entirely voluntary, and students could complete the questionnaire at their convenience within the designated timeframe.

The target population comprised medical students who had attended RO lectures and related coursework. Since the timing and structure of RO education varies across universities, an institution-specific distribution strategy was adopted:

- *Universities of Aachen, Cologne, and Düsseldorf*: Invitations were extended to students from semester 7 onwards, including those in the final year of clinical training (practical year; *praktisches Jahr*, PJ).
- *University of Bonn*: Invitations were sent to students from semester 6 onwards.

Radiation oncology was taught in varying intensities and teaching formats. At university A (Aachen), RO is integrated into the pathology curriculum, with 14 teaching units dedicated to RO. At universities B and C (Bonn and Cologne) it is part of an interdisciplinary module—cross-sectional subject 11 “imaging procedures, radiation therapy, radiation protection” (*Querschnittsbereich 11: Bildgebende Verfahren, Strahlenbehandlung, Strahlenschutz, QB 11*)—with four teaching units each. University D (Düsseldorf) offers RO as a standalone subject with 13 teaching units. Across these sites, RO teaching formats included traditional lectures, seminars, and practical sessions (collectively referred to as supplementary courses in this study).

### Recruitment strategy

A pilot questionnaire was developed by FL, MK, and PL. To ensure that the questions were neutral, clear, and easily comprehensible, a sample questionnaire was administered and pilot-tested among medical students and RO faculty members in C before the formal survey launch. Recruitment began in January 2022, starting with an email invitation sent to RO departments providing an overview of the study’s objectives, methodology, and significance. Student councils at all four universities were informed, and at ACD, the lead researcher (FL) personally met with representatives to explain the study. Social media channels (including student semester WhatsApp groups) and personal messages were used for additional outreach. At AC, faculty members directly emailed students and promoted the study during RO lectures. Monthly reminders were sent out to enhance recruitment.

### Survey description

The survey was developed based on an existing, validated evaluation tool used by the Faculty of Medicine, UoC, for assessing university teaching quality. The customized questionnaire focused on RO education and was enhanced by additional items to comprehensively capture student feedback.

The survey incorporated both quantitative and qualitative measures. Most items were rated using a five-point Likert scale (1 = “strongly disagree” to 5 = “strongly agree”). This scale is commonly used in educational research, as it facilitates clear distinctions between levels of agreement or satisfaction, aiding in statistical comparisons. Cronbach’s alpha was calculated to assess the internal consistency (reliability) of Likert scale items. Dichotomous (yes/no) questions explored students’ experiences, while open-ended questions allowed students to provide qualitative feedback regarding strengths, weaknesses, and suggestions for improvement.

Before starting the survey, participants received detailed study information and provided digital informed consent. The questionnaire consisted of 31 items, divided into seven thematic sections:

1. Demographic data (e.g., university, semester, gender).
2. Evaluation of radiation oncology lectures (11 items).
3. Evaluation of supplementary RO courses (9 items).
4. Perceived clinical relevance of course content (2 items).
5. Student interest in increasing RO teaching hours (2 items).
6. Overall satisfaction with RO education (1 item).
7. Qualitative feedback (praise, criticism, and suggestions; 3 items).

### Adaptive survey logic

The survey employed branching logic, dynamically adjusting the displayed questions based on previous answers. For example, students who had never attended an RO lecture were asked to specify their reasons for non-attendance, while those who experienced online learning due to the COVID-19 pandemic were asked to compare it with in-person instruction.

### Data analysis and statistical methods

The study employed a mixed-methods approach, integrating quantitative statistical analysis with qualitative content analysis. Inferential statistical analyses were not performed due to the relatively small sample size. Beforehand, it was calculated that a minimum of 128 responses were required for a meaningful analysis. The response rate was calculated as the ratio of received responses to the total number of students in the investigated semesters.

#### Quantitative analysis

Descriptive statistics (absolute frequencies, percentages, means, standard deviations) were computed. Statistical analysis was performed with the SPSS Statistics software package version 28 (IBM Corp., Armonk, NY, USA) for descriptive statistics.

#### Qualitative analysis

Open-ended responses were analyzed using thematic coding, following a standardized framework from the Humboldt University of Berlin for evaluating university teaching quality [28]. Two independent reviewers performed the coding to ensure inter-rater reliability. Any discrepancies were resolved through discussion via consensus with the research team to improve objectivity and minimize bias. Responses were categorized into five thematic groups:

1. Teaching quality and communication skills of instructors.
2. Teaching and learning methods.
3. Clinical and research integration.
4. Learning outcomes and assessment methods.
5. Positioning of RO within the medical curriculum.

### Ethical standards

The study protocol was approved by the Ethics Committee of the Faculty of Medicine, UoC (approval number: 20-165). The study adhered to the Declaration of Helsinki and followed institutional guidelines for research involving human participants. The questionnaire was provided on the basis of and in accordance with the regulations of DEGRO’s data protection conditions, too.

## Results

### Participants

A total of 193 questionnaires were collected, of which 152 were fully completed, resulting in an overall response rate of 4% based on an estimated total of approximately 3800 eligible students across the four universities. Overall, 41 incomplete responses were excluded to prevent duplicate entries and ensure data integrity. Of the respondents, 66% identified as female, with a mean semester of study of 8.7 (standard deviation [SD] = 1.6). Participant demographics and response rates (ABCD) are summarized in Table 1.

**Table 1:** Participant demographics (*n* = 152) and ABCD response rates.

Variable	Category	*n* (%)
Gender	Female	100 (66)
	Male	51 (33)
	Other	1 (1)
Semester of study	< 6th Semester	5 (3)
	6th Semester	10 (7)
	7th Semester	26 (17)
	8th Semester	31 (20)
	9th Semester	22 (15)
	10th Semester	41 (27)
	Practical year	17 (11)
Response rate	Aachen (A)	53 (7)
	Bonn (B)	21 (2)
	Cologne (C)	60 (5)
	Düsseldorf (D)	18 (1)
	Overall (mean)	(4)

As the survey link was disseminated publicly, responses were received from five students in lower semesters (< 6th) who had not yet reached the required eligibility criteria. Initially, these responses were analyzed separately to prevent potential bias. However, their feedback was ultimately integrated into the final dataset to ensure a more comprehensive representation of student perspectives.

The internal consistency of the survey instrument was assessed using Cronbach’s alpha. The reliability coefficient for lecture-related items was α = 0.71, while supplementary course-related items demonstrated high reliability, with α = 0.90.

### Evaluation of lectures in RO

Of the 152 students surveyed, 57% (*n* = 86) reported regularly attending RO lectures, while 30% (*n* = 46) attended occasionally, and 13% (*n* = 20) never attended. Among students who did not attend, the primary reasons cited included time constraints and a preference for alternative study methods. In total, 87% of students participated in lecture-based instruction to some extent; 66% (*n* = 100) of participants attended lectures exclusively online, using platforms such as Zoom due to the COVID-19 pandemic.

In contrast, 18% (*n* = 27) experienced solely in-person instruction, while 16% (*n* = 24) had a hybrid learning experience, combining both in-person and online formats. Concerns that digital teaching might lead to decreased attendance rates were not supported by the data. While 19% (*n* = 28) of students reported attending digital lectures less frequently than in-person classes, 38% (*n* = 57) attended at the same rate and 43% (*n* = 65) attended more frequently in the digital format.

**Fig. 1:**
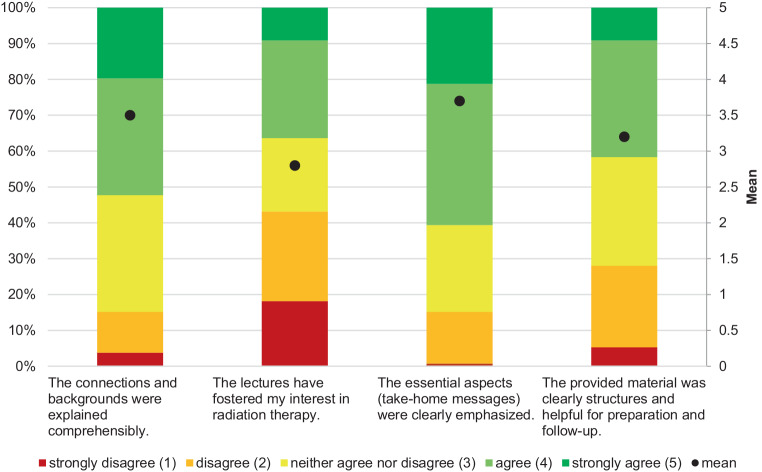
Evaluation of lectures in radiation oncology (*n* = 152). Students assessed lecture attendance patterns, didactic quality, engagement, clarity of key concepts, and quality of teaching materials

Furthermore, students were asked to evaluate the didactic skills of lecturers, the extent to which lectures fostered interest in RO, the clarity of key concept explanations, and the quality of the provided learning materials (Fig. 1).

### Evaluation of supplementary courses in RO (seminars, practical sessions)

Of the 152 participants, 61% (*n* = 92) reported the availability of supplementary courses, such as seminars and practical sessions. Among those with access, 85% (*n* = 129) participated, a rate comparable to lecture attendance (87%; *n* = 132).

The majority (51%) of supplementary courses were conducted digitally, reflecting the format used for lectures. However, a greater proportion of seminars and practical sessions were conducted exclusively in person (34%) as compared to lectures (18%).

Students rated small-group seminars and practical sessions as more engaging and effective than lectures. Among students advocating for additional semester hours, most preferred small-group seminars (42%) and practical training (39%) over traditional lectures (17%).

**Fig. 2:**
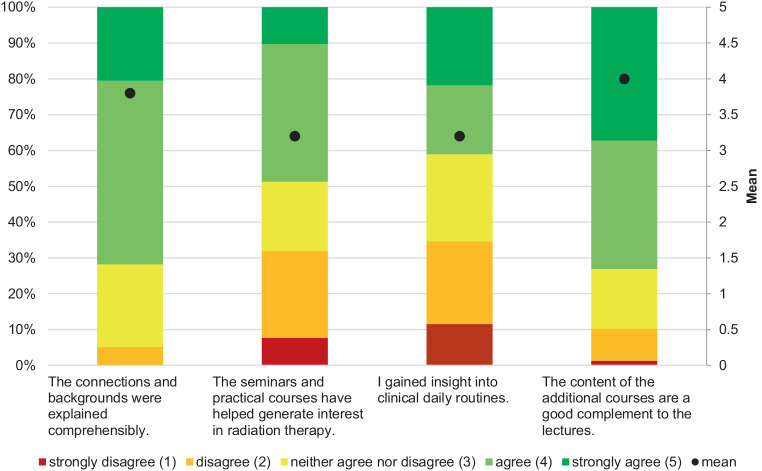
Evaluation of supplementary courses in radiation oncology, including seminars and practical sessions (*n* = 152). Participants rated the availability, participation, teaching format (digital/in-person), and perceived added value of these courses

To facilitate comparisons between lectures and supplementary courses, students were asked similar questions regarding course comprehensibility and whether the sessions fostered interest in RO. Additionally, participants assessed whether seminars and internships provided insights into clinical routines and how effectively they complemented lecture-based instruction (Fig. 2).

### Practical application, e.g., (digital) patient interviews, bedside teaching

Only 13% (*n* = 19) of the students reported having direct patient interaction in the field of RO. Instead, clinical examples played a central role in teaching, and 76% (*n* = 115) of students indicated that lecturers incorporated clinical case examples to support knowledge transfer.

### Future of radiation oncology education

The average number of semester hours (*Semesterwochenstunden*, SWS) allocated to RO education across the four universities was 9 (A: 14, D: 13, and B and C: 4). One SWS corresponds to a weekly class session of 45 min during a semester. To assess student preferences regarding the structure of future RO education, respondents were asked whether they would support an increase in allocated semester hours. Responses were split evenly, with 50% advocating for expansion and the remaining 50% opposed to expansion. Among students opposing an increase, no distinction was made between those favoring a reduction in teaching hours and those supporting the current allocation.

Regarding the preferred format for additional teaching hours, internships (42%) and seminars (39%) were the most favored options. In contrast, 17% of students preferred additional lecture-based instruction (multiple selections were allowed).

### Student ratings of RO education

Students rated RO education at their respective universities on a German school grading scale ranging from 1 (highest) to 6 (lowest), yielding a mean score of 2.8. Among students who assigned a poor rating (grades 4, 5, or 6), 59% expressed a desire for increased RO credit hours, compared to 45% among students rating the course more favorably (grades 1–3). No statistically significant differences were observed between universities, indicating that variations in credit hours did not influence overall course evaluations.

Students who regularly attended lectures provided significantly higher ratings (mean: 2.6) compared to occasional attendees (3.0) and non-attendees (3.5). Occasional attendees graded the course at 3.0. Regular attendees assigned a significantly higher rating of 2.6. Additionally, students who reported an increased interest in RO due to the course assigned more favorable ratings (2.3) than those who did not feel encouraged (3.3).

No significant differences were found between exclusively in-person and fully digital teaching formats. However, students who attended hybrid lectures (both in-person and online; 16%) assigned an average rating of 2.5, compared to 2.9 among students who experienced a single teaching format (either fully in-person or fully digital).

### Qualitative analysis: praise, criticism, and suggestions for improvement

A total of 217 free-text responses were provided, comprising 63 expressions of praise, 83 critical remarks, and 71 suggestions for improvement. The responses were categorized into eight predefined thematic groups. Praise, criticisms, and suggestions are summarized in Table 2.

**Table 2:** Praise, criticism, and suggestions to improve the radiation oncology educational program—qualitative analysis (free-text responses)

Category	Praise	Criticism	Suggestions
Didactic aids/materials	Sufficient materials	Insufficient supplementary materials for content exploration, preparation, or review	*(More) materials for preparation and review* (*e.g., script with key terms and procedures)*
	Materials accessible online after the course	Unclear and disorganized slides	*Better structured and more organized slides*
	Structured materials	Unstructured lecture organization	Timely upload of lecture materials
Engagement/motivation	–	Failure to foster interest in the subject	–
Student interaction/communication	–	Direct questions possible only in live format	–
Structure/organization/planning	–	Unclear learning objectives and take-home messages	Clearly define learning objectives and take-home messages
		Unstructured online platform	
Teaching and learning formats	Substantial student interaction Small group seminars and practical sessions are effective Visits to RO facilities	Lack of interaction Traditional lectures Online teaching: PowerPoint with audio recording instead of live instruction Limited face-to-face instruction	*Clinical ward visits, including procedural inspections* *(More) small group seminars and practical sessions* More interactive teaching, less frontal instruction Face-to-face teaching Incorporate quiz-based learning Offer an online tool Introduce problem-based learning (PbL)
Research and practical relevance	Use of clinical examples	Lack of clinical examples No practical relevance No patient contact	*(More) patient contact opportunities* *Presentation of the daily routine of radiation therapists* *(More) clinical case studies* (More) practical relevance/clinically contextualized topics
Academic rigor and learning outcomes	–	Inadequate explanation of fundamentals	–
		Limited long-term learning gain	
		No recollection of RO	
Curriculum structure	Interdisciplinary approach (oncology, pathology, physics) RO in preclinical curricula	Insufficient interdisciplinary teaching Scattered teaching schedule (courses and exams in different semesters) Not enough semester hours Lack of coordination between modules (e.g., seminar topics not covered in lectures) Repetitive content	*Consolidation of all modules within one semester* Enhance interdisciplinarity Improved coherence among modules (e.g., explaining devices based on physics principles) Clearer differentiation from other subjects, e.g., radiology, nuclear medicine, physics

The aspects mentioned at least five times are in italics, the remaining suggestions are listed in descending order of frequency. Some categories contain predominantly critical comments, reflecting areas of specific student concern. Thematic grouping follows a standardized evaluation framework used in structured teaching evaluations

*RO *radiation oncology

### Comparison of results with DEGRO recommendations [25]

Student preferences largely aligned with DEGRO’s recommendations, particularly regarding increased practical relevance through patient contact and clinical case studies. However, notable discrepancies emerged, including students’ preference for a clearer distinction between RO and other disciplines. Table 3 highlights areas of agreement and partial or no alignment between student suggestions and DEGRO guidelines.

**Table 3:** Comparison and alignment of student recommendations with DEGRO guidelines.

Suggestions	Students’ recommendations	DEGRO’s recommendations [25]
*Curriculum structure*		
More SWS^a^	Partially congruent	Congruent
Integration of RO in preclinical phase	Congruent	Congruent
Expansion of QB^a^	Partially congruent	Congruent
Enhance interdisciplinarity	Congruent	Congruent
Distinct separation of RO from other subjects^b^	Congruent	Not addressed
All modules in one semester^a^	Partially congruent	Not addressed
Elective courses	Congruent	Congruent
*Practical relevance*		
More patient contact, e.g., through bedside teaching	Congruent	Congruent
Clinical case studies	Congruent	Congruent
Course on medical communication	Congruent	Congruent
*Teaching format*		
Expansion of digital/hybrid teaching^a^	Partially congruent	Congruent
More small-group teaching	Congruent	Congruent
Less traditional lectures, more interaction	Congruent	Congruent
(Guided) independent study	Congruent	Congruent
Modern teaching formats (e.g., PbL, quiz) ^a^	Congruent	Partially congruent
Emphasis on learning objectives^b^	Congruent	Not addressed
Development of key knowledge	Congruent	Congruent

^a^Aspects where recommendations were discrepant between students and DEGRO

^b^Aspects where recommendations partially differed between students and DEGRO

*SWS* semester hours,* RO* radiation oncology*, QB* cross-sectional block*, PbL* problem-based learning

## Discussion

This study provides valuable insights into the current state of RO education at four German medical schools and their associated university hospitals. The findings highlight both strengths and areas for improvement, particularly concerning curriculum structure, teaching formats, and practical exposure. While students acknowledged the benefits of small-group learning and hybrid teaching models, they also identified significant shortcomings, including the absence of structured learning objectives, limited patient contact, and insufficient differentiation between RO and related disciplines (e.g., radiology, nuclear medicine). These results are consistent with previous studies indicating that early exposure, interactive teaching methods, and structured curricula play a crucial role in enhancing students’ knowledge and interest in RO [29]. While the small sample size warrants cautious interpretation and highlights the need for further longitudinal research to assess the generalizability of our findings, this study also emphasizes that structured student surveys are a valuable tool for aligning teaching content and formats with learner needs and for informing curriculum development.

One of the central challenges identified in this study is the lack of structured learning objectives, an issue that has been highlighted in previous research on medical curricula [30, 31]. ABCD students reported difficulties in identifying key takeaways and understanding the daily routine of a radiation oncologist, which could limit their engagement with RO and reduce its perceived relevance [28]. While Dapper et al. highlight the importance of expanding key knowledge areas, future DEGRO recommendations could further enhance clarity by incorporating clearly defined learning objectives and competency-based benchmarks to better align with student needs and ensure a more structured learning experience [25]. In this context, a clearer prioritization of learning content—differentiating between core curriculum elements and supplementary knowledge—could help to guide students more effectively and improve the overall educational framework.

Kandiko Howson et al. reported similar findings, where unclear curricular goals were linked to lower student satisfaction and engagement [32]. A previously published study by the medical education research team at the University of Cologne by Linde et al. found that students struggle to distinguish RO from related fields, such as radiology and nuclear medicine, leading to confusion regarding the specialty’s role in patient care, which is further exacerbated by the shared structural framework within QB 11 in Germany, although the interdisciplinary approach should be positively emphasized [15]. These results suggest that improving curricular transparency and defining specific competencies could significantly enhance learning outcomes. A structured feedback mechanism should be introduced, allowing student representatives to actively participate in curriculum planning committees.

The study also underscores the importance of interactive teaching formats, aligning with competency-based medical education models. Seminars and practical sessions were rated more effective in terms of generating interest in RO compared to lectures. Students expressed strong support for PbL, simulation-based training (SBT), and quiz-based learning, which are known to enhance problem-solving skills and student motivation [33–35]. A multicenter study conducted by the Radiation Oncology Educational Collaborative Study Group (ROECSG) demonstrated that structured workshops and hands-on practical sessions significantly improve students’ understanding of RO concepts [36]. Similarly, research on interactive contouring modules found that such methods increased students’ awareness of radiotherapy processes and side effects, reinforcing the need for modernized, practically oriented teaching approaches [32]. However, it should also be acknowledged that experiential learning formats—such as patient-centered teaching, interdisciplinary tumor boards, and extensive practical sessions—require increased personnel, time, and organizational resources, which vary significantly between faculties. While DEGRO’s recommendations serve as a gold standard, their implementation may be hindered by such disparities: not all faculties have the staff or structural conditions needed to fully realize these goals. Our findings further reveal that students frequently experience unmet needs—such as clearer differentiation between RO, radiology, and nuclear medicine—that may be underappreciated in guideline-based planning. This gap highlights that curriculum delivery (i.e., how content is structured and taught) may weigh more heavily than the number of teaching hours alone. In sum, innovative formats are strongly supported by students, but their successful integration depends not only on educational intent, but also on their feasibility within local institutional frameworks.

In line with previous studies, students in this study favored hybrid learning models, with those who participated in both in-person and online instruction rating RO education more favorably than those who experienced only one teaching modality [7, 15, 37, 38]. This finding aligns with broader research demonstrating that blended learning strategies optimize engagement by accommodating different learning styles [39]. Digital learning, which extends beyond merely recording lectures, e.g., in the form of virtual reality-based simulations, offers flexibility and allows students to revisit course materials at their own pace. However, students also emphasized the importance of interactive elements, structured feedback, and faculty engagement [40–43]. Although our data were collected during the pandemic-driven shift to digital formats (2020–2022), some changes—such as hybrid teaching models and the expectation of flexible access to on-demand lectures—may persist beyond COVID-19. These findings underscore the need for well-structured hybrid teaching approaches that integrate interactive digital modules with essential in-person training to ensure both accessibility and engagement.

A further key issue concerns curriculum structure and interdisciplinary integration. While the use of a longitudinal distribution of RO teaching is supported, students in this study preferred consolidating all RO-related courses into a single semester, arguing that time gaps between lectures, seminars, and practical sessions contribute to knowledge loss. This discrepancy is inconsistent with findings in evidence-based RO medical education [44, 45].

Research in curriculum development suggests that improved integration across disciplines—e.g., linking radiation oncology with medical physics, anatomy, or biology through case-based learning—could enhance conceptual clarity and interdisciplinary understanding [46, 47]. This aligns with findings that young oncology professionals in Germany see a strong need for interdisciplinary collaboration despite practical barriers [48]. Another promising approach to fostering interdisciplinary learning is the implementation of a student tumor board. This format, recently piloted, engages students in simulated multidisciplinary oncology meetings, enhancing their understanding of how different specialties (including RO) collaborate in patient care [49]. These findings highlight the need for careful consideration of longitudinal curriculum design, balancing student preferences with pedagogical best practices while also taking into account the specific context, objectives of the medical education program, and learner expectations [50]. A further important aspect to consider is whether an early introduction to RO can serve not only as a connecting element to the (preclinical) curriculum but also foster long-term enthusiasm for the field among medical students [51].

The study findings further confirm the strong correlation between student engagement and positive course evaluations. Students who regularly attended lectures assigned higher ratings to RO education, supporting the argument that active participation enhances learning outcomes [28]. These trends are consistent with previous studies in different fields such as internal medicine or radiology, where greater exposure to the field correlated with increased interest in the specialty [52, 53]. Furthermore, mentorship was identified by Marsiglio et al. as a critical factor in guiding students toward careers in RO, yet formal mentorship opportunities remain limited [54]. Enhancing early mentorship programs could help to close this gap and inspire more students to pursue a career in RO, both during medical school and in postgraduate training [9, 52, 55]. Furthermore, strategically placed initiatives, such as social media campaigns in medical education, can help to reshape the image of RO and dispel common urban myths [56].

### Seven recommendations for enhancing RO education

Based on the findings of this study, the following key recommendations, actionable steps, and focal takeaways are proposed to enhance RO education and address the student-identified gaps from our survey (Table 2):

1. Implement hybrid teaching models for greater accessibility
  *Recommendation*: Hybrid teaching formats should be formally integrated into RO education, as they provide flexibility and maintain high participation rates.
  *Actionable step*: Faculties should balance prerecorded lectures, live online discussions, and in-person sessions to optimize engagement.
2. Improve learning materials for better structure and comprehension
  *Recommendation*: Teaching materials should be revised to be more structured and accessible.
  *Actionable step*: Institutions should provide well-organized lecture slides, summary sheets, and digital resources to facilitate student preparation and review.
3. Adopt interactive and case-based teaching methods
  *Recommendation*: Transition from traditional lectures to PbL and interactive case discussions to enhance student engagement.
  *Actionable step*: Develop standardized case studies that integrate clinical decision-making exercises into the curriculum, e.g., case-based tumor boards.
4. Expand small-group and hands-on learning opportunities
  *Recommendation*: Increase small-group seminars and practical sessions, which students find more effective than traditional lectures.
  *Actionable step*: If SWS are expanded, prioritize practical, discussion-based learning rather than increasing lecture hours.
5. Strengthen practical relevance through direct clinical exposure
  *Recommendation*: Increase bedside teaching, patient interactions, and hands-on experience with radiation therapy procedures.
  *Actionable step*: Introduce mandatory clinical rotations in RO departments to expose students to real-world applications.
6. Establish and communicate clearer learning objectives for RO
  *Recommendation*: Clearly define RO as a distinct discipline and improve differentiation from radiology and nuclear medicine.
  *Actionable step*: Develop standardized competency-based learning objectives, ensuring consistency across medical schools.
7. Expand semester hours and consider mandatory key sessions
  *Recommendation*: Increase RO teaching hours, as students who attended more courses showed greater interest and gave higher evaluations.
  *Actionable step*: Implement mandatory attendance for core RO sessions while offering elective advanced modules and RO research tracks and mentorship for interested students.

By implementing these evidence-based improvements, medical faculties and RO departments can enhance the quality of RO education, increase student interest, and ultimately support the development of future radiation oncologists. Expanding the study to include a larger, multi-institutional sample would enhance the generalizability of these findings and provide a more comprehensive understanding of best practices for RO education.

### Limitations

Several methodological limitations may impact the interpretation of this study’s findings. One primary concern is the low response rate of 4%, which may not fully represent the target population. However, this rate could be underestimated due to limitations in survey distribution, as it remains unclear whether all eligible students received the questionnaire. Consequently, the actual response rate might be higher than reported. We also explicitly acknowledge the possibility of self-selection bias: for example, students with strong opinions about RO (either positive or negative) might have been more likely to respond, which could skew the results.

The absence of inferential statistical analyses prevents identification of statistically significant relationships between the examined variables. While such analyses would have been desirable to strengthen the validity of the findings, they were not feasible due to the limited sample size and response rate. As a result, the study relies solely on descriptive statistics, which further restricts the generalizability of the results. Due to our sample size, we may not have detected subtle benefits of a broader curriculum (e.g., better factual knowledge), so this question warrants further study, too. To enhance the robustness of future research, a larger and more representative sample should be obtained to allow for inferential statistical testing. Given this limitation, the reported trends should be interpreted with caution, as observed differences between groups may not necessarily indicate causal relationships.

Additionally, the categorization of qualitative responses into major themes introduces a potential interpretation bias. Despite using predefined categories and employing two independent reviewers for classification, subjective judgment in assigning free-text responses remains a limitation.

A notable discrepancy was observed in the reported availability of supplementary courses. Although all universities offered these courses, only 61% of respondents acknowledged them. This inconsistency suggests that some students may not have recognized specific events as being part of the RO curriculum or may have forgotten about them. This issue is likely exacerbated by the integration of RO into cross-sectional blocks with other disciplines (e.g., radiology, nuclear medicine, pathology), making course attribution more challenging.

Lastly, the COVID-19 pandemic significantly impacted teaching methods, forcing a shift from in-person to digital instruction. This transition may have reduced practical skill development and interactive learning opportunities, leading to a possible decline in student engagement and understanding of clinical applications.

Despite these limitations, the study provides valuable perspectives on student experiences and recommendations for improving radiation oncology education.

## Conclusion

This study highlights the need to adapt RO education to student needs, modern teaching approaches, and competency-based learning. Drawing on student feedback, we propose seven targeted recommendations, including clearer learning objectives, more interactive and clinically relevant teaching, and an improved curriculum structure. Implementing these student-informed strategies may substantially enhance RO education and align it more closely with national standards. Strengthening collaboration between faculties and professional societies like DEGRO will be essential to ensuring a standardized and future-oriented RO education that fosters engagement and prepares the next generation of radiation oncologists.

## Abbreviations

ABCD: Aachen, Bonn, Cologne, Düsseldorf
ÄApprO: Licensing Regulations for Physicians; *Ärztliche Approbationsordnung*
DEGRO: German Society of Radiation Oncology; *Deutsche** Gesellschaft für Radioonkologie*
PbL: Problem-based learning
PJ: Practical year
QB 11: Cross-sectional subject 11 “imaging procedures, radiation therapy, radiation protection”
RO: Radiation oncology
SWS: Semester hours
UoC: University of Cologne
